# Morphological Analysis of Bronchial Arteries and Variants with Computed Tomography Angiography

**DOI:** 10.1155/2017/9785896

**Published:** 2017-07-04

**Authors:** Claudia Nallely Esparza-Hernández, Juan Manuel Ramírez-González, Rolando Alberto Cuéllar-Lozano, Rodolfo Morales-Avalos, Carla Sofía González-Arocha, Brenda Martínez-González, Alejandro Quiroga-Garza, Ricardo Pinales-Razo, Guillermo Elizondo-Riojas, Rodrigo Enrique Elizondo-Omaña, Santos Guzmán-López

**Affiliations:** ^1^Facultad de Medicina, Human Anatomy Department, Universidad Autónoma de Nuevo León, Monterrey, NL, Mexico; ^2^Hospital Universitario “Dr. José Eleuterio González”, Radiology and Imaging Division, Universidad Autónoma de Nuevo León, Monterrey, NL, Mexico

## Abstract

The aim of our study was to determine the prevalence of anatomical variants of bronchial arteries using computed tomographic angiography in a population of northeastern Mexico. An observational, transversal, descriptive, comparative, retrospective study was performed using 139 imaging studies of Mexican patients in which we evaluated the following parameters from the left and right bronchial arteries: artery origin, branching pattern, arterial ostium, vertebral level of origin, diameter, and mediastinal trajectory. The anatomies of the bronchial arteries were similar in both genders, except distribution for vertebral origin level (*p*  0.006) and the diameter (*p*  0.013). Left and right arteries were similar, except for the mediastinal trajectory in reference to the esophagus (*p* < 0.001) as well as the arterial diameter (*p* < 0.001) and lumen diameter.

## 1. Introduction

Massive hemoptysis is a life-threatening clinical event [1]. Its main etiology is secondary to rupture of the bronchial arteries and rarely from other systemic arteries or the pulmonary artery [2]. Chronic inflammatory lung diseases (e.g., tuberculosis) produce hypertrophy and fragility of the bronchial arteries, which can lead to rupture and bleeding [1].

The most effective nonsurgical treatment for massive hemoptysis is the embolization of bronchial arteries [1–13]. Its location by imaging studies is the most important step to control bleeding [2]. They are defined as any artery which provide irrigation to the main bronchus originating between T5 and T6 of the descending aorta; those originating out of this region are considered ectopic [14]. Knowledge of anatomical variants could reduce the time of diagnosis and intervention, patient radiation, and embolization material costs. It could also avoid complications such as ischemia from nonvisible anastomoses [3, 15–18].

The anatomical variants of the bronchial arteries have been reported in cadaveric studies, angiography, and computed tomography angiography (CTA); the latter was considered the most suitable method [14, 15, 18–21]. Cauldwell et al. (1948) [22] were the first to describe patterns of branching of the bronchial arteries and their classification is currently the most widely used [1, 15]. Yener et al. (2015) studied the branching patterns by computed tomography and reported that the most frequent variation (24.03%) corresponded to a left bronchial artery originating directly from the thoracic aorta and the right bronchial artery originating from the intercostobrachial trunk (type II Cauldwell) [15]. Other studies have described the origin of the bronchial arteries, without reporting its full pattern, which limits the analysis of the results [14, 17, 18]. Other variables have been evaluated by computed tomography, such as ostium of the bronchial arteries in the aorta [23], the vertebral levels origin [14, 15, 18, 24], path through the posterior mediastinum [18], and diameter [14, 19, 25, 26]. To our knowledge, there are no studies that evaluate all of these variables in the same population.

The objective of our study was to analyze the arterial origin, branching pattern, location ostium, vertebral level of origin, mediastinal trajectory, and diameter of the bronchial arteries using CTA.

## 2. Materials and Methods

We performed a retrospective, observational, cross-sectional descriptive study, in which 297 angiograms were evaluated by CTA from a Mexican population, carried out in the Radiology and Imaging Division of the Hospital Universitario “Dr. José Eleuterio González.” Consecutive cases were selected between January 1, 2013, and December 31, 2015, from patients who were 18 years old or older, including those with suspected pulmonary, pleural, or heart pathologies. Studies from patients younger than 18 years were excluded. Pathologies that did not allow the proper evaluation of one or both bronchial arteries were eliminated (158 studies).

CTA studies were performed using a 64-slice CT (General Electric CT99 Light Speed VCT) Software 2978195VCT, with a 0.4 s rotation of helical acquisition, 40 mm detector coverage, 120 Kv with a 400 mAs; slice thickness of 1.25 mm, 0.984 : 1 mm/rot Pitch, and a 40 to 50 cm FOV. All patients were injected with intravenous iodinated contrast medium (Ultravist 370, Bayer, Germany) at a dose of 1 to 2 ml/kg with a 3.5 ml/second injection rate. The data obtained were transferred and analyzed in Osirix Lite station in its version of 32 bits for Mac devices on OS X10.8, using multiplanar reformatting (MPR) with maximum intensity projection (MIP) and volume rendering (VR). To perform the measurements, a range of window WW: 400 and WL: 40 (modified when needed for dynamic evaluation) was used in a standardized manner for all patients taking into account the internal diameter of each artery evaluated.

Imaging studies were evaluated by an experienced radiologist with prior training in the use of software and knowledge of the most commonly accepted anatomical classification (Cauldwell et al. [22]).

The evaluation of the bronchial arteries was performed according to the following variables: origin, branching patterns, location ostium, vertebral level source, diameter, and mediastinal trajectory in reference to the esophagus and the main bronchi.

The origin of the each bronchial artery could be independent, a common trunk or an intercostobrachial trunk. In turn, these arteries of origin could originate from the thoracic aorta, the aortic arch, the subclavian arteries, internal thoracic artery, or the thyrocervical trunk. A bronchial common trunk was considered when two or more bronchial arteries originated from the primary vessel before contacting the main bronchus. The aortic arch was considered to end and the thoracic aorta to begin, at the lower border of the T4 vertebra.

**Figure 1:**
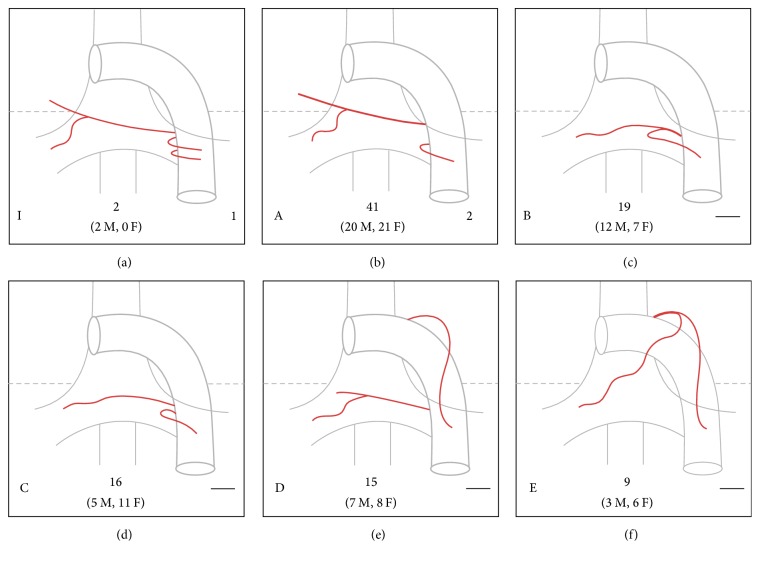
Branching patterns of bronchial arteries. Left lower letter indicates the corresponding pattern described in our study and the right lower number corresponds to the classification by Cauldwell et al. (a line means that the pattern was not included in the Cauldwell classification). Center lower number represents the total frequency with male and female reported between parenthesis (M = male; F = female). (a) shows the pattern I of our study, type 1 of Cauldwell, corresponding to “typical” anatomy of bronchial arteries described in medical textbooks. (b–f) show the most frequent branching patterns found in our study (patterns A to E, resp.).

After establishing the origin of each of the bronchial arteries, their trajectory continued until contact with the corresponding main bronchus to determine their side (left or right) and branching pattern. Patterns found were ordered according to their frequency and alphabetical letters (A–L) were designated for identification. Single frequency patterns were considered atypical and were grouped in the L category and assigned Arabic numerals pattern (L1–L24) (Figure 1 and Figure 1 of the supplementary files in Supplementary Material available online at https://doi.org/10.1155/2017/9785896).

**Figure 2:**
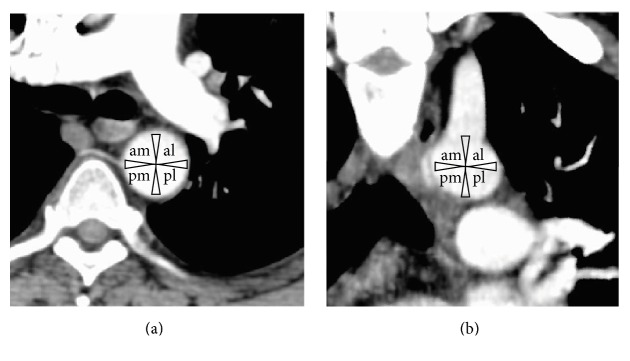
Ostium location methodology for bronchial arteries. (a) is cross-section of a thoracic CT scan when the origin is from the thoracic aorta, and (b) is a coronal section of a thoracic CT scan when the origin is from the aortic arch. Assessment of the ostium location of each bronchial artery at its origin is completed using the methodology described by Battal et al. [23]. am = anterior-medial, al = anterior-lateral, pm = posterior-medial, and pl = posterior-lateral.

Ostium location for bronchial arteries originating from the thoracic aorta and aortic arch was determined using the methodology by Battal et al. [23] as shown in Figures 2(a) and 2(b), respectively.

**Figure 3:**
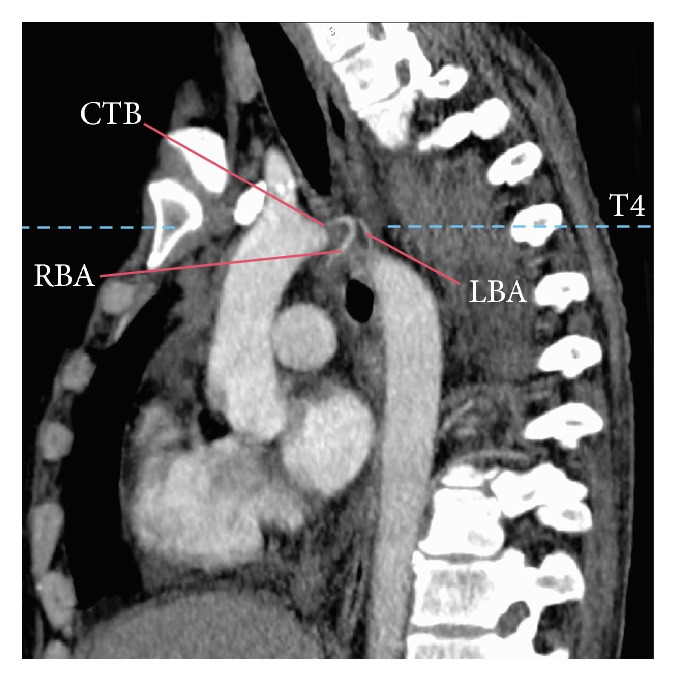
Bronchial artery branching pattern evaluation. A sagittal section of a thoracic CT scan which is used to evaluate the vertebral level of the bronchial artery origin of the bronchial arteries is shown (dashed line). A branching pattern of the bronchial arteries from a common trunk (one right and one left) originating from the aortic arch is also shown. CTB = common trunk of the bronchial arteries, RBA = right bronchial artery, and LBA = left bronchial artery.

The vertebral level origin was evaluated by drawing a horizontal line from the origin of each of the bronchial arteries to interconnect with the vertebrae (C7-T7) (Figure 3). Arteries originating between T5 and T6 were considered orthotopic, and as ectopic those arising beyond these levels [14, 18, 23, 27].

Position of each bronchial artery in relation to its mediastinal trajectory (left or right) was documented. The position (anterior or posterior) of the bronchial artery in relation to the main bronchus was determined by observing the point of first contact between the two structures.

The diameter of all bronchial arteries, common trunks, and intercostobrachial trunks involved was documented at the origin of the vessel. All measurements were reported in millimeters and were stored in a database for subsequent statistical analysis.

Subsequent to morphological and morphometric analysis, a statistical comparison between sides (left versus right) and gender (men versus women) for all variables was performed.

### 2.1. Statistical Analysis

Statistical analysis was performed using the computer program SPSS version 20.0 for Windows XP. Measures of central tendency and dispersion tests were obtained by descriptive statistics for qualitative data. Nonparametric Kolmogorov-Smirnov to analyze the distribution of data was performed. Binomial tests of a sample were performed to determine whether there was a difference between the number of men and women and the number of left and right bronchial arteries. Subsequently, Mann–Whitney *U* tests were performed to determine whether there was a difference between each of the qualitative variables regarding side (left versus right) and gender. Likewise, Student's *t*-test for independent samples was performed to assess statistical differences regarding diameter in relation to gender and side. A *p* of <0.05 was considered as significant.

### 2.2. Ethical Considerations

This study was approved by the Ethics Committee and the Research Committee of the Universidad Autónoma de Nuevo León, Facultad de Medicina, with the registration number AH14-005. No patient was radiated nor received contrast for the purposes of this study. There was no financial or commercial gain in the realization of this study, so the authors declare no conflicts of interest.

## 3. Results

We evaluated 139 angiograms by CTA (73 men; 66 women) from a population of Mexican patients. The mean age was 49.90 years ± 18.62 with a range between 18 and 90 years. Men had a mean age of 47.40 ± 19.05 years with a range between 19 and 90, while women had a mean of 52.67 ± 17.86 with a range between 18 and 85 years. Gender groups were distributed homogeneously (*p* = 0.215).

A total of 315 bronchial arteries were identified, of which 209 (66.34%) were orthotopic, 95 (54.54%) left, and 114 (45.45%) right sided. We also found 106 (33.65%) ectopic bronchial arteries, of which 69 (65.09%) were left and 37 (34.90%) right.

We identified between 2 and 5 bronchial arteries distal to the origin per patient, with higher prevalence for the left side than the right; however this difference was not statistically significant (*p* = 0.499). Men also had a higher number of bronchial arteries than women but it was not statistically significant (*p* = 0.650).

All bronchial arteries were evaluated according to their origin, branching patterns, ostium location, vertebral level of origin, lumen diameter, and mediastinal trajectory in reference to the esophagus and the main bronchus.

### 3.1. Artery Origin

**Figure 4:**
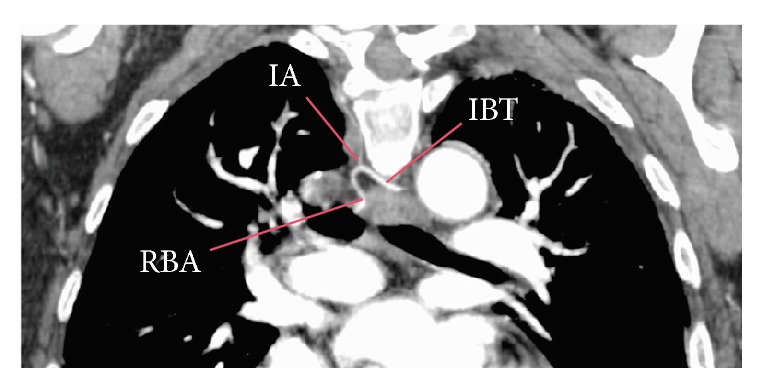
Branches of an intercostobrachial trunk. This contains a right bronchial artery originating from an intercostobrachial trunk shown in a coronal section. IBT = intercostal-bronchial trunk, RBA = right bronchial artery, and LBA = left bronchial artery.

Bronchial arteries originated from the thoracic aorta (74.60%), aortic arch (23.49%), and other vessels (1.90%). In the aorta they emerged as independent branches (40.95%), branches from a common trunk (29.84%) (Figure 3), or branches of an intercostobrachial trunk (27.30%) (Figure 4). The origins of other vessels (1.90%) were 2 left bronchial arteries from the left subclavian artery, one right of the right thyrocervical trunk, one right and one left from a common trunk of the left thyrocervical trunk, and one left from the left internal thoracic artery. Frequencies of origin are shown in Table 1. There was no significant difference between arterial origins regarding the side (*p* = 0.293) or on gender (*p* = 0.354).

### 3.2. Branching Pattern

We identified 35 different branching patterns (Figure 1 and Figure 1 of the supplementary files). Patterns “A” to “K” were presented with more than one frequency and patterns “L (1–24)” as single cases. The most common branching patterns were the “A,” “B,” “C,” “D,” and “E,” respectively (Figure 1). Twenty-four atypical patterns (L [1–24]) were found (Figures 3 and 4 of the supplementary files). Patterns “A,” “C,” “D,” and “E” occurred more frequently in women, while patterns “B” and “F” to “J” were more frequent in men, with pattern “K” equally presented in both genders (Figure 2 of the supplementary files). Men had a higher prevalence of atypical patterns with 14 cases and 10 cases in women. Branching patterns and the frequency are listed in Figure 1 and Figure 1 of the supplementary files.

### 3.3. Ostium

The location of the ostium depended on the artery origin. In the bronchial arteries originating from the thoracic aorta, the location of the ostium was found mainly in the anterior-medial portion (58.29%), followed by the medial portion (13.19%) and anterior-lateral portion (10.63%). For these arising from the aortic arch, the location of the ostium was found mainly in the superomedial portion (60.81%), followed by inferior-medial (14.86%) and superior-lateral (13.51%) (Table 2). There was no significant difference in the location ostium between the left and right bronchial arteries (*p* = 0.398) and no significant difference between genders (*p* = 0.872).

### 3.4. Vertebral Level

All bronchial arteries originated between C7 and T7 vertebral levels. Between T5 and T6 66.34% of bronchial arteries originated, 54.54% were right sided, and 45.45% were left, 55.98% were men, and 44.01% women. Above the vertebral level T5, 32.69% originated, of which 34.95% were right and 65.04% left, 48.54% corresponded to men and 51.45% to women. Below T6, the remaining 0.95% originated, 33.33% were right, 66.66% were left, 66.66% were men, and 33.33% women (Table 3).

Men had higher incidence of orthotopic bronchial arteries (55.98%) but were similar to women in ectopic bronchial arteries (49.05% versus 50.94%, resp.) (Table 3). A statistically significant difference in the distribution of vertebral origin level between genders (*p* = 0.006) was identified; however there is no difference for this parameter between arteries on both sides (*p* = 0.997).

### 3.5. Mediastinal Trajectory

The left bronchial arteries passed in 96.95% of cases to the left of the esophagus and 3.04% to the right of the esophagus. The right bronchial arteries passed in 56.29% of cases to the right of the esophagus and 43.70% to the left of the esophagus (Table 4). A significant difference was established between both sides in the mediastinal trajectory regarding the esophagus (*p* < 0.001), but there was no significant difference between genders (*p* = 0.943).

Both the left and the right bronchial arteries came in contact with the corresponding main bronchus predominantly on the posterior surface (70.15%). The trajectory in relation to the main bronchus was the same on both sides (*p* = 0.318) and in both genders (*p* = 0.527) (Table 4).

### 3.6. Diameter

In both genders, the luminal diameter of the bronchial arteries had a mean of 1.62 ± 0.29 mm with a range of 1.14 to 2.97 mm (the minimum and maximum values were both found in the left bronchial arteries) (Table 5). However, in most cases, the right bronchial arteries showed larger diameters than the left (*p* < 0.001). Significant difference was also evident between genders (*p* = 0.013), as men had larger diameters than women.

### 3.7. Bronchial Artery Common Trunk

Forty-six common trunks of bronchial arteries were identified in 46 patients, of which 28 (60.86%) originated from the thoracic aorta, 17 (36.95%) from the aortic arch, and one (2.17%) originated from a left thyrocervical trunk. For those originating from the thoracic aorta, the ostium location was primarily in the anteromedial portion (53.57%), and for those from the aortic arch the ostial location was primarily in the superomedial portion (64.70%). In the thoracic aorta originated, 60.71% originated at the vertebral level T5, and in the aortic arch 76.47% at the vertebral level T4. The common trunk that originated from the left thyrocervical trunk has a vertebral level of C7-T1. The mean diameter was 1.82 ± 0.35 mm (range 1.19 to 2.69 mm).

The common trunks gave rise to 94 bronchial arteries, of which 43 were right (45.74%) and 51 left (54.25%). In 40 cases (86.95%), it was the origin for one left bronchial artery and one right bronchial artery. In 4 cases (8.69%) it branched one right and two left bronchial arteries, and in 2 cases (4.34%) two left bronchial arteries (Figure 1 and Figure 1 of the supplementary files). Its trajectory through the mediastinum passed to the left of the esophagus, although not all arteries followed this path, since some branched the corresponding bronchial arteries before along the esophagus. None of the common trunks came in direct contact with a main bronchus.

### 3.8. Intercostobrachial Trunk

A total of 83 intercostobrachial trunks were found in 82 patients, of which 77 (93.90%) originated in the thoracic aorta and 6 (7.22%) in the aortic arch. The ostium localization was found predominantly in the anterior-medial portion (54.54%) in the ones originating from the thoracic aorta and in the inferior-medial portion (50%) for the ones originating in the aortic arch. The vertebral level was T5 (62.33%) and T4 (100%), respectively, with a mean luminal diameter of 1,99 ± 0.40 mm (range 1.32 to 3.21 mm).

The intercostobrachial trunks gave rise to a total of 86 bronchial arteries, of which 80 (93.02%) were right and 6 (6.97%) left (Figure 1 and Figure 1 of the supplementary files). In most cases (96.38%) it gave rise to one right bronchial artery and one posterior intercostal artery; in two cases (2.40%) it is divided into one left bronchial artery and one posterior intercostal artery; in one case (1.20%) it originated from one left bronchial artery, one right bronchial artery, and posterior intercostal artery; and in one case (1.20%) it branched into one right bronchial artery, two left bronchial arteries, and posterior intercostal artery. Most trunks branched before passing next to the esophagus; therefor they were not included in the mediastinal trajectory analysis. None of the intercostobrachial trunk came in contact with a main bronchus.

## 4. Discussion

Our study is the first to comprehensively assess the anatomy of the bronchial arteries, common trunks, and intercostobrachial trunks using CTA in the same population. We are the first to report all branching patterns found and perform statistical analysis for all parameters according to gender and side. We showed that in our community there are anatomical variations in terms of numbers, arterial origin, branching pattern, vertebral level of origin, mediastinal trajectory, and diameter. The knowledge of these is essential for embolization procedures in the treatment of massive hemoptysis.

There is a high frequency of ectopic origin, reported between 13 and 74% [14, 15, 18, 23, 28], which is why it is important to consider the location of a bleeding bronchial artery before and during the embolization procedure. In our study we found a frequency of 66.34% of orthotopic bronchial arteries and 33.65% of ectopic. The frequency with which bronchial arteries are affected by hemoptysis processes relates to the nature of their origin [4], reason why previous authors describe them; these tend to be branches of the thoracic aorta and aortic arch, emerging, in order of frequency as independent branches of a common trunk, an intercostobrachial trunk, and other arteries other than the aorta [15, 18, 23], similar to what was found in our study.

In patients with massive hemoptysis requiring urgent arterial embolization, the lack of familiarity with the most common variants of bronchial arteries may be an important factor in the success or failure of the intervention procedure [23]. The branching patterns of bronchial arteries were initially described by Cauldwell et al. in 1948 (Table 6) and, since then, they have been used as reference for bronchial artery embolization in cases of massive hemoptysis and for anatomical studies [14, 15, 18–21, 23]. Our study found that the most common branching pattern corresponds to the type A (type II Cauldwell), which coincides with the most frequently reported by previous authors [1, 15, 22]. It is followed by type B and C patterns, similar to that reported in previous studies [1, 15]; however, neither of these was described by Cauldwell et al. [22]. In addition, pattern “I” of our study (type I Cauldwell [Figure 1]), presented in the most widely used anatomy books [29–31], does not represent the totality of existing anatomical patterns nor its actual frequency according to recent literature [15]. The study of bronchial artery branching patterns in other populations is necessary to determine their prevalence.

Previous authors have reported a predominant anterior-medial location of the ostium in the thoracic aorta [15, 18, 23] and superior-medial in the aortic arch [23], similar to our findings. Previous studies evaluated the arterial diameter at a proximal point to the origin, because when it is above 2 mm, it is considered an indicator of a pathological bronchial artery [25].

As observed in our results, other authors report a smaller luminal diameter for the left bronchial arteries; however this might be because there are more artery origins for the left bronchus than the right [14, 15, 18, 23]. Consequently this difference in diameter may be due to anatomical physiology, rather than pathology.

In some cases massive hemoptysis may be treated surgically [32] that is why its location in the posterior mediastinum is important [18]. Previous studies have described that the trajectory of the left bronchial arteries is to the left of the esophagus and the right bronchial arteries to the right of the esophagus, both predominantly coming in contact with their respective main bronchus on the posterior surface [15, 18], similar to our results.

Some studies report a higher number of right bronchial arteries compared to the left [18, 23]; however our results indicate a higher amount of left bronchial, as reported by Cauldwell et al., although this difference was not statistically significant [22]. We believe that this discrepancy may be because these studies reported cases of patients with a single bronchial artery, which, most often, is right sided. In our study we found 5 cases with only right arteries and 2 with only left arteries, which were excluded from the sample. We believe that it is important to research the alleged “only bronchial artery” because it has been described that the bronchial arteries are vital for the proper functioning of the airway and lungs, and damage by erroneous embolization could lead to problems like ischemia and necrosis of mucosa, chronic lung problems, among others [16]. During interventional angiography, if three bronchial arteries are not identified on the same side of hemoptysis, there is higher risk of recurrence of bleeding. The third artery might not be identified either because it does not exist or because it was not identified.

Digital subtraction angiography could possibly help identify small caliber bronchial arteries, not visible through CTA. However, this type of imagining study would limit the morphological description and relationship to adjacent structures. A limitation of our study was the omission of the bronchial arterial length because it was not possible to observe the full length of the vessel in a single projection and reformatting was needed for full view. Also, the diameter of bronchial arteries may vary between our sample population and a healthy one due to the physiopathology, because we included patients with pulmonary, pleural, and/or heart pathologies.

## 5. Conclusion

Our study is the first to assess comprehensively by computed angiotomography the anatomy of the bronchial arteries, common trunks, and intercostobrachial trunks in the same population. We are the first to report all branching patterns found and perform statistical analysis for all parameters according to gender and side. The anatomy of the bronchial arteries is similar in both genders, except vertebral level origin and diameter, and similar in relation to side (left versus right) except the mediastinal trajectory reference to the esophagus and lumen diameter. The data presented in this study demonstrated that the branching patterns of bronchial arteries and their frequencies reported in the literature do not coincide with results from a Mexican population. Further studies using higher resolution imaging techniques are needed to study the morphology of the bronchial arteries.

## Supplementary Material

Supplementary Figure 1: This contains the 35 branching patterns of bronchial arteries. Left lower letter indicates the corresponding pattern described in our study and the right lower number corresponds to the classification by Cauldwell et al. (a line means the pattern was not included in the Cauldwell classification). Center lower number represents the total frequency with male and female reported between parenthesis (M = Male, F = Female).
Supplementary Figure 2: This contains a special case of the branching pattern “K” of the bronchial arteries.
Supplementary Figure 3: This contains a special case of the branching pattern “L2” of the bronchial arteries.
Supplementary Figure 4: This contains a special case of the branching pattern “L21” of the bronchial arteries.

## Figures and Tables

**Table 1:** Origin of bronchial arteries (*n* = 315).

Origin	Side		Total (M, F)
	LBA (M, F)	RBA (M, F)	
Thoracic aorta			
Independent	74 (38, 36)	23 (11, 12)	97 (49, 48)
IBT	6 (5, 1)	74 (38, 36)	80 (43, 37)
CTB	32 (20, 12)	26 (17, 9)	58 (37, 21)
Aortic arch			
Independent	29 (17, 12)	3 (1, 2)	32 (18, 14)
IBT	0 (0, 0)	6 (4, 2)	6 (4, 2)
CTB	19 (7, 12)	17 (6, 11)	36 (13, 23)
Others	4 (3, 1)	2 (2, 0)	6 (5, 1)
*Total (n)*	*164*	*151*	*315*

RBA = right bronchial artery, LBA = left bronchial artery, IBT = intercostal-bronchial trunk, CTB = common trunk of the bronchial arteries, M = male, and F = female.

**Table 2:** Location of the ostium of the bronchial arteries (*n* = 315).

Ostium location	Independent		IBT		CTB		Others		Total
	RBA	LBA	RBA	LBA	RBA	LBA	RBA	LBA	
Thoracic aorta									
Anterior-medial	15	48	42	0	15	17			**137**
Anterior-lateral	2	12	1	0	3	7			**25**
Posterior-medial	1	3	13	2	0	0			**19**
Posterior-lateral	0	1	0	0	0	0			**1**
Anterior	4	8	0	0	5	5			**22**
Medial	1	2	18	4	3	3			**31**
Aortic arch									**0**
Superior-medial	3	17	1	0	11	13			**45**
Superior-lateral	0	4	0	0	3	3			**10**
Inferomedial	0	6	3	0	1	1			**11**
Superior	0	1	0	0	1	1			**3**
Inferior	0	0	0	0	1	1			**2**
Medial	0	1	2	0	0	0			**3**
Others							2	4	**6**
*Total *	*26*	*103*	*80*	*6*	*43*	*51*			*315*

RBA = right bronchial artery, LBA = left bronchial artery, IBT = intercostal-bronchial trunk, and CTB = common trunk of the bronchial arteries.

**Table 3:** Vertebral level origin of the bronchial arteries (*n* = 315).

Vertebral level	RBA (M, F)	LBA (M, F)	Total (M, F)
C7-T1	2 (2, 0)	1 (1, 0)	**3 (3, 0)**
T2	0 (0, 0)	1 (1, 0)	**1 (1, 0)**
T3	2 (2, 0)	4 (3, 1)	**6 (5, 1)**
T3-T4	2 (1, 1)	5 (3, 2)	**7 (4, 3)**
T4	22 (8, 14)	41 (19, 22)	**63 (27, 36)**
T4-T5	8 (4, 4)	15 (6, 9)	**23 (10, 13)**
T5	75 (38, 37)	56 (27, 29)	**131 (65, 66)**
T5-T6	6 (3, 3)	9 (6, 3)	**15 (9, 6)**
T6	33 (20, 13)	30 (23, 7)	**63 (43, 20)**
T6-T7	1 (1, 0)	0 (0, 0)	**1 (1, 0)**
T7	0 (0, 0)	2 (1, 1)	**2 (1, 1)**
Total (*n*)	151 (79, 72)	164 (90, 74)	**315 (169, 146)**

RBA = right bronchial artery, LBA = left bronchial artery, C = cervical, T = thoracic, M = male, and F = female.

**Table 4:** Mediastinal trajectory of the bronchial arteries in relation to the esophagus and main bronchus (*n* = 315).

Artery	Esophagus			Main bronchus		
	Left	Right	*p*	Left	Right	*p*
LBA	159	5	0.001	53	111	0.318
RBA	66	85		41	110	
*Total*	*225*	*90*		*94*	*221*	

RBA = right bronchial artery and LBA = left bronchial artery.

**Table 5:** Diameters of the bronchial arteries.

		Mean ± SD (mm)	Range (mm)	Total, *n*
Female	LBA	1.49 ± 0.26	1.14–2.97	74
	RBA	1.67 ± 0.30	1.22–2.55	72
	Total	1.58 ± 0.29	1.14–2.97	146
Male	LBA	1.58 ± 0.21	1.19–2.32	90
	RBA	1.75 ± 0.34	1.31–2.93	79
	Total	1.66 ± 0.29	1.19–2.93	169
Total	LBA	1.54 ± 0.24	1.14–2.97	164
	RBA	1.71 ± 0.32	1.22–2.93	151
	Total	1.62 ± 0.29	1.14–2.97	315

SD = standard deviation, RBA = right bronchial artery, and LBA = left bronchial artery.

**Table 6:** Cauldwell classification.

Type	Pattern	Frequency
1	Two left independent of the thoracic aorta and one right originated from intercostobrachial trunk	40.6%
2	One left independent of the thoracic aorta and one right originated from intercostobrachial trunk	21%
3	Two left independent of the thoracic aorta and two right (one independent of the thoracic aorta and one originated from intercostobrachial trunk)	20%
4	One left independent of the thoracic aorta and two right (one independent of the thoracic aorta and one originated from intercostobrachial trunk)	9.7%
