# Supplementary material for: Morphological Analysis of Bronchial Arteries and Variants with Computed Tomography Angiography
Source: Biomed Res Int. 2017 Jul 4;2017:9785896. doi: 10.1155/2017/9785896 (PMC5514344; doi:10.1155/2017/9785896)
Supplement: Supplementary file 1 — Supplementary Figure 1: This contains the 35 branching patterns of bronchial arteries. Left lower letter indicates the corresponding pattern described in our study and the right lower number corresponds to the classification by Cauldwell et al. (a line means the pattern was not included in the Cauldwell classification). Center lower number represents the total frequency with male and female reported between parenthesis (M = Male, F = Female). Supplementary Figure 2: This contains a special case of the branching pattern “K” of the bronchial arteries. Supplementary Figure 3: This contains a special case of the branching pattern “L2” of the bronchial arteries. Supplementary Figure 4: This contains a special case of the branching pattern “L21” of the bronchial arteries. [file 9785896.f1.pdf]

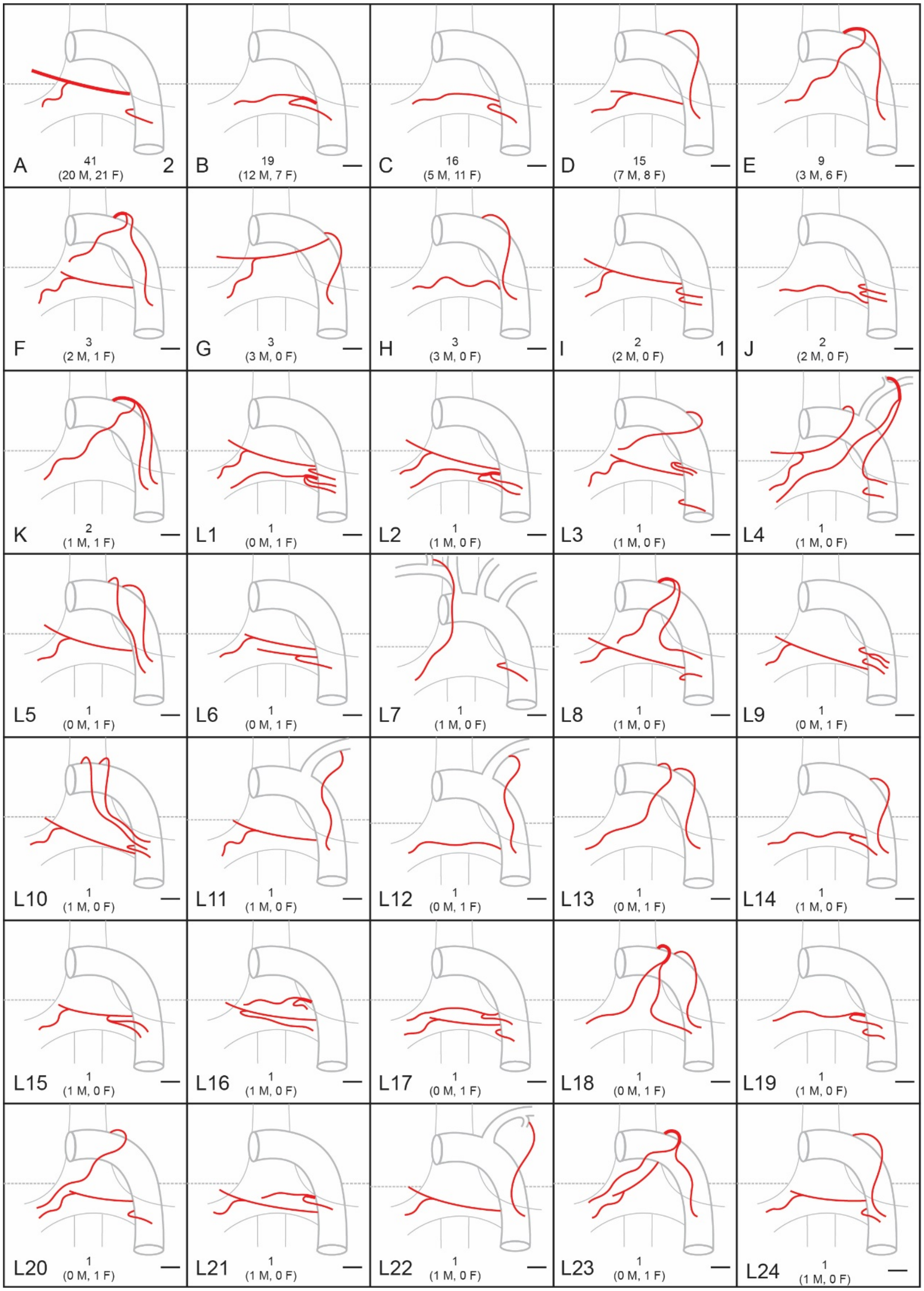

Supplementary files

Figure 1

This contains the 35 branching patterns of bronchial arteries. Left lower letter indicates the corresponding pattern described in our study and the right lower number corresponds to the classification by Cauldwell *et al.* (a line means the pattern was not included in the Cauldwell classification). Center lower number represents the total frequency with male and female reported between parenthesis (M = Male, F = Female).

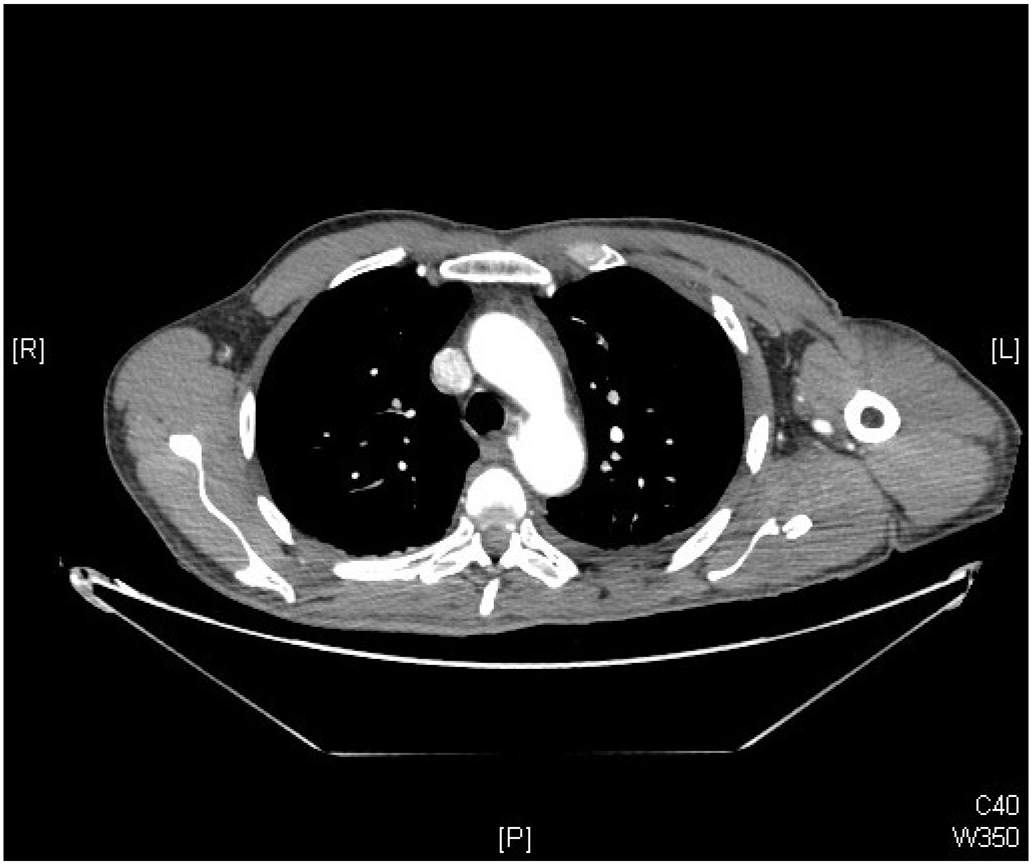

Supplementary files

Figure 2

This contains a special case of the branching pattern "K" of the bronchial arteries.

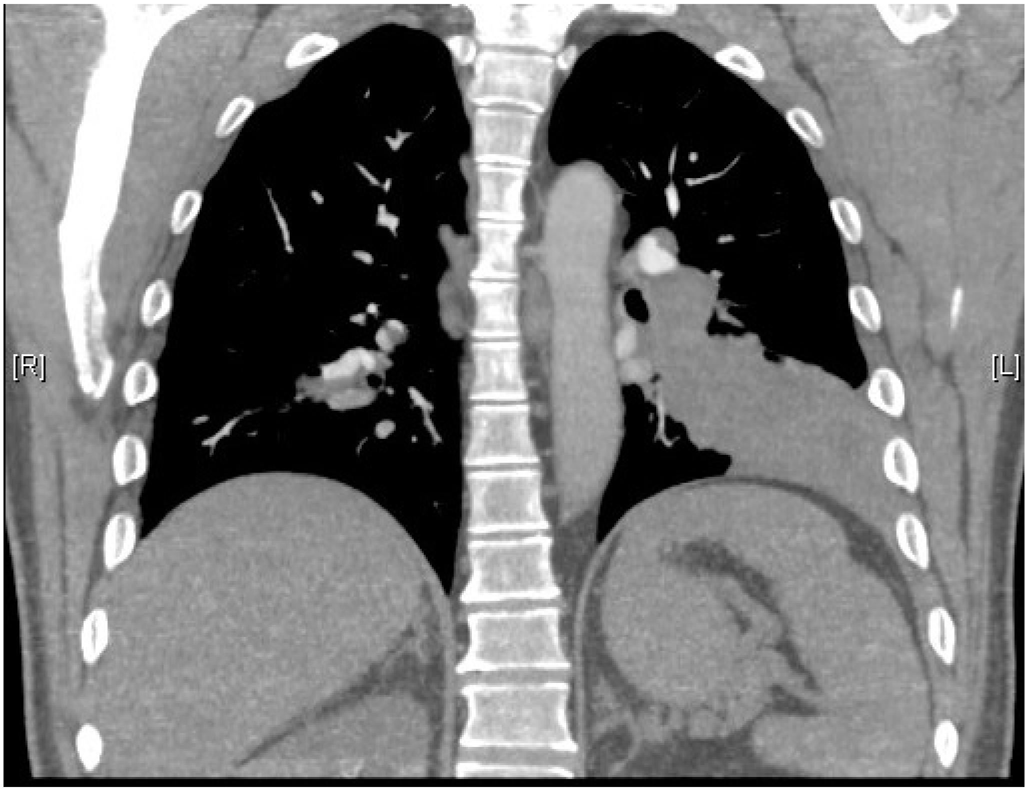

Supplementary files

Figure 3

This contains a special case of the branching pattern “L2” of the bronchial arteries

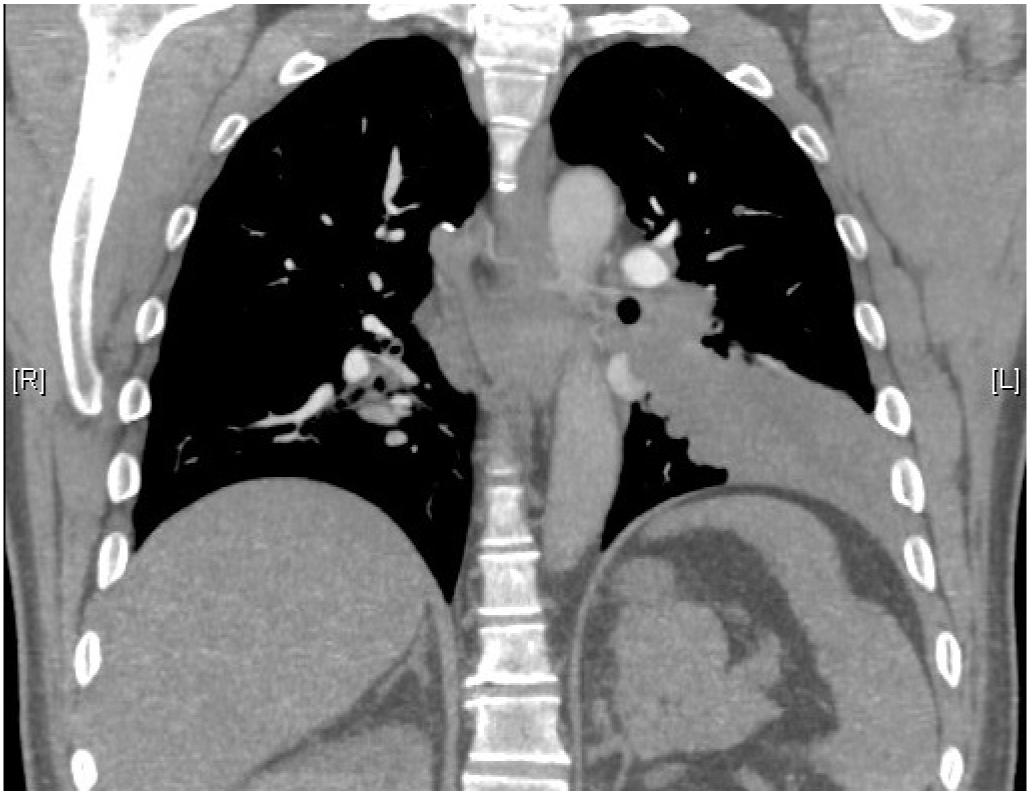

Supplementary files

Figure 4

This contains a special case of the branching pattern “L21” of the bronchial arteries.
